# Comments on species divergence in the genus *Sphaerium* (Bivalvia) and phylogenetic affinities of *Sphaerium nucleus* and *S*. *corneum* var. *mamillanum* based on karyotypes and sequences of 16S and ITS1 rDNA

**DOI:** 10.1371/journal.pone.0191427

**Published:** 2018-01-23

**Authors:** Romualda Petkevičiūtė, Virmantas Stunžėnas, Gražina Stanevičiūtė

**Affiliations:** P. B. Šivickis Laboratory of Parasitology, Nature Research Centre, Vilnius, Lithuania; Duke University Marine Laboratory, UNITED STATES

## Abstract

Chromosome, 16S and ITS1 rDNA sequence analyses were used to obtain reliable diagnostic characters and to clarify phylogenetic relationships of sphaeriid bivalves of the genus *Sphaerium*. The species studied were found to be diploid, with modal number 2n = 28 in *S*. *nucleus* and 2n = 30 in *S*. *corneum* var. *mamillanum*. Small, biarmed, C- negative B chromosomes were found in all studied populations of both species. Karyological and molecular markers revealed no differences between *S*. *corneum* s. str. and *S*. *corneum* var. *mamillanum*. No intraspecific differences were found in the basic karyotype of *S*. *nucleus*. Molecular analyses, however, uncovered three genetically distinct ITS1 lineages: one comprised of samples from Lithuania, Slovakia, and Russia, another from Czech, and a third from Ukraine. Additionally to known 16S haplotype from Ukraine, three new 16S haplotypes of *S*. *nucleus* were detected: one in the samples from Lithuania and Russia, one in Slovakian and one in Czech population. In the ITS1 phylogenetic tree, all branches of *S*. *nucleus* clustered in one clade. In the 16S phylogenetic tree, however, the haplotype of Czech *S*. *nucleus* formed a separate branch, distant from three other haplotypes of *S*. *nucleus*. Molecular results indicate that in the context of the Evolutionary Species Concept the *S*. *nucleus* morphospecies may represent a complex of separate taxa, however referring on the Biological Species Concept the genetic lineages could represent the intraspecific variability.

## Introduction

The cosmopolitan bivalve family Sphaeriidae represents one of the most widespread molluscan groups, inhabiting different freshwater habitats [1–3]. Estimation of sphaeriid species diversity has been greatly hampered by the highly variable shell morphologies exhibited by many taxa and the lack of reliable morphological traits for species differentiation [4–5]. Different taxonomic significance has been attributed to all levels of morphological variation by different taxonomic schools (see comments in [3,6–8]. Therefore, the taxonomic status of species within the traditional genus *Sphaerium* Scopoli 1777 and intrageneric grouping have generated considerable discussion for over a century. Based on the last taxonomic revision [3], the genus is represented in Europe by seven valid species. The most variable member of the genus is the type species *S*. *corneum* (L., 1758). Because of wide shell variability, several forms or varieties of the species have been distinguished. *Sphaerium nucleus* (Studer, 1820) is usually considered an intraspecific variety of *S*. *corneum* by Western specialists [2,9]. However, some conchological and anatomical characters to support the distinctness of *S*. *nucleus* were provided by Korniushin [3,10], with the shape of nephridium considered the most reliable of them. Nevertheless, anatomical characters are not widely used in sphaeriid taxonomy, and *S*. *nucleus* still is poorly known due to confusion with *S*. *corneum*. It is believed that the geographic range of the species comprises the major part of Europe [3], but only quite recently *S*. *nucleus* was reliably recorded in some Central European countries [11–14] and in Britain [15–16]. The exact geographic range of *S*. *nucleus* needs to be evaluated on the basis of new diagnostic characters. *Sphaerium corneum* var. *mamillanum* is considered an intraspecific variation by West-European malacologists [2,17] and a distinct species in Russian publications [10,18]. After a comprehensive morphological analysis, Korniushin [3] concluded that *S*. *corneum* var. *mamillanum* could not be definitely separated from typical *S*. *corneum*. Nevertheless, the problem of the taxonomic status of these two forms is still not conclusively resolved.

In cases where traditional taxonomy gives problematic results, species distinctness and the phylogenetic relationship of certain forms may be supported using karyological and/or molecular data. Unfortunately, the number of karyologically studied sphaeriid taxa is still very limited and the data for many of them are incomplete. Among the species that have been examined, highly polychromosomal nuclei are the rule, with chromosome numbers ranging to above 200 (see review in [19–20]). Prior to this study, only three sphaeriid species were known to be diploid: Palaearctic *S*. *corneum* and *S*. *solidum* and Nearctic *S*. *rhomboideum* [19,21–22]. Previous attempts to karyotype *S*. *nucleus* in order to find species-specific karyological characters and to compare it to *S*. *corneum* were unsuccessful [23].

This study is the first to characterize the mitotic chromosomes of *S*. *nucleus* and *S*. *corneum* var. *mamillanum*. We describe the karyotypes of *S*. *nucleus* obtained from three different populations in Central Europe and of *S*. *corneum* var. *mamillanum* from one population in Estonia using conventional karyometric analysis and C-banding. We also use molecular markers based on the nuclear ITS1 and mitochondrial 16S ribosomal gene fragment sequences that have been recently developed for numerous Holarctic sphaeriid species, for phylogenetic reconstructions [22,24–25]. These two regions of rDNA of *S*. *nucleus* and *S*. *corneum* var. *mamillanum* were sequenced from different populations, and the resulting alignments were used for comparative phylogenetic analyses to obtain species-specific markers.

## Materials and methods

Samples of *S*. *nucleus* were collected from three locations in Central Europe: in South Slovakia (48°25´32´´ N; 20°01´34´´ E, the sampling place indicated by Košel [12]), in Czech, South Moravia (48°44´58´´ N; 17°00´14´´ E, the sampling place indicated by Korinkova [11]), and in Lithuania from a marshy coast of Lake Terpežys (55° 15' 29.69" N; 25° 53' 51.17" E) in the Labanoras Regional Park. This species should be considered comparatively rare in Lithuania, as a number of favourable habitats were checked for its presence during 2006–2009, but the species was found only in the above-mentioned location. One specimen of *S*. *nucleus* was received from Russia (Moscow region) and used for comparative DNA analysis. Samples of *S*. *corneum* var. *mamillanum* were collected from the stream between Lake Liinjarv and Lake Suurjarv (57°43'35.64" N; 26°55'41.00" E) in Estonia. Also, further samples of *S*. *corneum* s. str. were collected from two water bodies in Estonia, Lake Mustjarv (57°56'6.41" N; 27°20'23.76" E) and River Vaike-Emajogi (57°59'8.64" N; 26° 2'55.28" E), and used for molecular analysis. The specimens were identified on conchological characters suitable for species identification according to Korniushin [3;10]. It was found that shell pore density is one of the most reliable diagnostic characters for preliminary differentiation of *S*. *nucleus* and *S*. *corneum*. According to the International Union for Conservation of Nature (IUCN) information there are no known conservation actions known for *S*. *nucleus* and *S*. *corneum*, and none are considered necessary. The populations are thought to be stable [26–27]. No permissions are required for their collection and further use for research. The field-collected species were sampled in free access water bodies, where no permission is needed. Voucher specimen shells from each of these samples have been deposited in the collection of the P.B. Šivickis Laboratory of Parasitology, Institute of Ecology of Nature Research Centre.

Brooding animals were found at the time of collection. For karyological analysis, whole intact living animals were incubated in 0.01% colchicine in well-water during 3 to 5 h. The bodies were removed from the shells under a dissecting microscope and treated for 50–60 min in distilled water for hypotony. The fixation was made in three changes (20 min each) of a freshly prepared fixative of ethanol-acetic acid (3: 1). Chromosome preparations were made with a cell suspension air-drying technique [21]. Each slide was made from the tissues of a single individual. Slides were stained in 4% Giemsa-Romanowski dye in phosphate buffer (pH 6.8) for 30–40 min. Chromosomes in suitable metaphases were counted and the best spreads were photographed using an Olympus BX51 light microscope supplied with a digital camera. The lengths of the short and long arms of chromosomes were measured in ten karyotypes from different individuals obtained from each population. Data analysis was performed with an Excel macro-program. Means and standard deviations of the absolute and relative lengths (100 x absolute chromosome pair length divided by the total length of the haploid complement) and the centromeric index (100 x length of the short arm divided by the total chromosome length) were calculated for each pair of chromosomes. Terminology relating to the centromere position follows that of Levan et al. [28], but a binary terminology was adapted when the 95% confidence limits of the centromeric index mean covered two chromosome categories. Data were analysed using the independent two-sample Student’s *t* test, and the results were considered significant when P<0.05. C-banding was carried out according to the Sumner [29] modified method, i.e., slides were treated with saturated Ba(OH)2 for 15 min, briefly washed in distilled water, 0.2 N HCl, distilled water again, incubated in 2 x SSC (0.3 M NaCl, 0.03 M Na3C6H5O7) for 90 min at 60° C, and stained for 1 h in a 5% Giemsa solution buffered to pH 6.8.

Total DNA for molecular analysis was isolated from the tissues of the same specimens used for cytogenetic studies according to the protocol of Stunžėnas et al. [22]. A nucleotide fragment ~480 bp of the mitochondrial large ribosomal subunit (16S) DNA was amplified using primers 16Sar (5’-CGC CTG TTT ATC AAA AAC AT-3’) and 16Sbr (5’-CCG GTC TGA ACT CAG ATC ACG T-3’) according to Palumbi [30]. An entire nuclear internal transcribed spacer 1 (ITS1) sequence (~560 bp) was amplified following the protocol of Stunžėnas et al. [22] and using primers from White [31] annealing to flanking regions of 18S and 5.8S genes; these primers were, respectively, 18SWF (5’-TAA CAA GGT TTC CGT AGG TG-3’) and 5_8_SWR (5’-AGC TRG CTG CGT TCT TCA TCG A-3’). The PCR product was purified and sequenced in both directions at Macrogen Inc. (Seoul, Korea). Sequence confirmation was accomplished by comparing complimentary DNA strands. Editing of the DNA sequences, contig assembly, and the alignment of the consensus sequences were carried out using the software program Sequencher 4.7 (Gene Codes Corporation).

Additional sequences were downloaded from GenBank and included in the phylogenetic analysis: *Sphaerium nucleus* from Ukraine (AY093537, AY093573), *S*. *corneum* (AY792316, AY792317, AY792319, AY792320, AY792321, AY093535, AF152037), *S*. *solidum* (FJ874903, FJ874904, GU123690, FJ874907, FJ874908, FJ874909), *S*. *rhomboideum* (AF152038, AY093538), *S*. *occidentale* (AF152046, AY093542), *S*. *baicalense* (AY093534). Sequences of *Pisidium dubium* (AF152027, AY093533) and *P*. *variabile* (AF152030, AY093530) were included as the outgroup taxa.

For phylogenetic analyses, the sequences of ITS1 dataset were aligned using ClustalW [32] with an open gap penalty of 15 and gap extension penalty of 6.66. Multiple Sequence Alignment Software MAFFT version 7 [33] with iterative refinement method of G-INS-i were used to align sequences of 16S dataset, because MAFF produced better parsimony-informative alignment comparing with ClustalW (34 vs 32 parsimony informative sites). The best-fit model of sequence evolution for phylogenetic analysis was estimated using jModeltest v. 0.1.1 software [34]. Ambiguously aligned positions were excluded from phylogenetic analysis. Nucleotide by nucleotide distance between sequences was estimated in MEGA6 [35] using model No. of differences with pairwise deletion of gaps/missing data and inclusion of all substitutions (transitions and transversions). Maximum likelihood phylogenetic trees were obtained and analysed using MEGA6. Branch support was estimated by bootstrap analyses with 1000 replicates. The phylogenetic trees were obtained using general time reversible model with a gamma distribution of rates and a proportion of invariant sites (GTR + G + I) for both the ITS1 and the 16S gene datasets. Gamma shape and number of invariant sites were estimated from the data.

## Results

### Karyotype of *Sphaerium nucleus*

**Fig 1:**
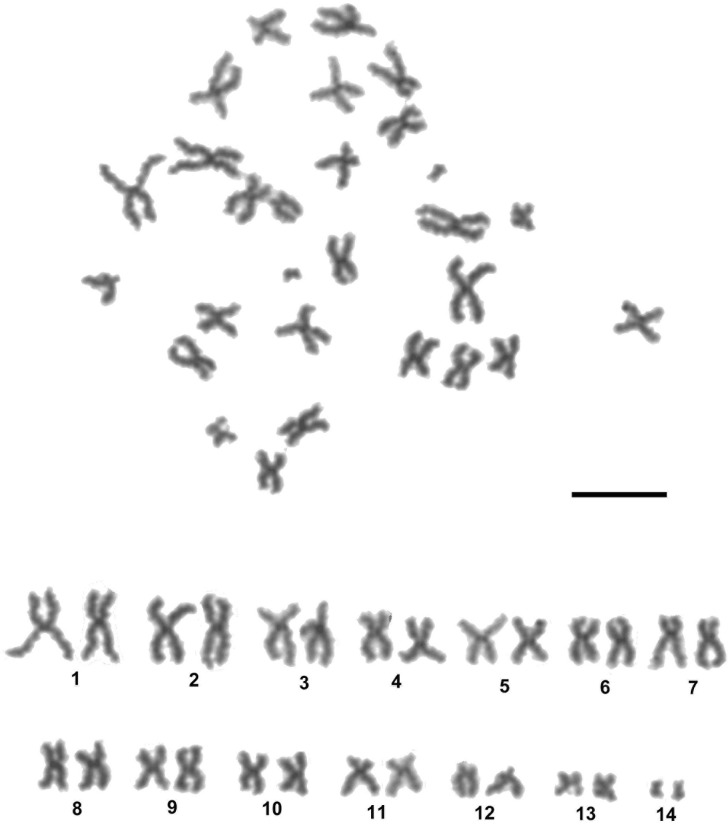
A mitotic metaphase chromosome spread and the karyotype of *Sphaerium nucleus*, 2n = 28. Scale bar = 10 μm.

A total of 304 mitotic metaphase spreads from 33 individuals of *S*. *nucleus* (15 from Lithuania, 11 from Slovakia, and 7 from Czech) were analysed, and the modal diploid chromosome number of 2n = 28 was revealed (Table 1). A representative karyotype is shown in Fig 1. The chromosomes in the karyotype show a regular decrease in size, except for the last pair, which is strikingly smaller than the others. Table 2 indicates the absolute length, relative length, centromeric index (CI), and classification of the chromosome pairs in each of the three populations. The chromosomes ranged in size from 1.16 μm to 9.47 μm. The mean total length of the haploid complement (TCL) ranged from 61.83 μm in the Slovak population to 79.34 μm in the Lithuanian population. Differences in the absolute length of chromosomes may be partially accounted for by different chromosome condensation on the slides studied. The karyotype consisted of all biarmed, metacentric, meta-submetacentric, and submetacentric chromosomes. The lowest CI value was estimated in chromosomes pair 12, and they were classified as submetacentric. Within the karyotype of *S*. *nucleus*, pairs of homologous chromosomes could be distinguished by their morphology, except for pairs 6 and 7, and pairs 9 and 10, both sets of which had similar relative lengths and centromeric indices (see Table 2). Comparative study revealed no significant (P<0.05) interpopulation differences in relative lengths and CI values of the corresponding chromosomes of the basic complement.

**Table 1:** Chromosome numbers in mitotic metaphases of *Sphaerium nucleus* and *S*. *corneum* var. *mamillanum* from different populations in Europe (S, Slovak, L, Lithuanian, C, Czech, E, Estonian population).

Species /population origin/ number of specimens	Chromosome number (main complement and supernumerary Bs) in mitotic metaphase						
	27	28	30	32	34–35	36	4n
*S*. *nucleus* /L/ 15	8 (4.32%)	150 (81.1%)	-	-	4 (2.16%)	20 (10.8%)	3 (1.62%)
*S*. *nucleus* /S/ 11	2 (2.6%)	48 (62.3%)	-	15 (19.5%)	1 (1.3%)	11 (14.3%)	-
*S*. *nucleus* /C/ 7	4 (9.52%)	31 (73.8%)	-	6 (14.3%)	-	-	1 (2.38%)
*S*. *corneum* var mamillanum /E/ 10	-	-	60 (69.77%)	2 (2.33%)	20 (23.25%)	4 (4.65%)	-

**Table 2:** Measurements (mean±SD) and classification of modal diploid (A) chromosomes of *Sphaerium nucleus* (S, Slovak, L, Lithuanian, C, Czech population).

Chromosome number		Absolute lenght (μm)	Relative length (%)	Centromeric index	Classification*
1	S	7.86±1.34	12.70±0.97	47.15±2.48	m
	L	9.47±2.03	11.86±0.65	44.90±2.88	
	C	8.80±1.76	12.14±0.77	45.27±2.78	
2	S	6.95±1.18	11.19±0.56	45.23±2.74	m
	L	8.43±1.61	10.61±0.60	42.35±3.61	
	C	8.00±1.04	11.15±0.50	44.97±3.24	
3	S	5.36±0.81	8.67±0.58	37.59±1.39	m-sm
	L	7.19±1.41	9.05±0.51	38.06±1.53	
	C	6.60±1.16	9.15±050	37.33±3.07	
4	S	4.93±0.70	7.96±0.43	44.70±2.70	m
	L	6.44±1.22	8.11±0.37	43.32±3.85	
	C	5.91±0.94	8.20±0.29	40.86±3.18	
5	S	4.76±0.62	7.71±0.47	42.45±3.99	m
	L	6.32±1.16	7.97±0.37	41.63±4.14	
	C	5.68±0.76	7.91±0.31	40.15±3.57	
6	S	4.55±0.67	7.36±0.50	44.68±3.62	m
	L	5.97±1.13	7.51±0.20	44.58±2.25	
	C	5.40±0.76	7.51±0.26	44.38±2.97	
7	S	4.38±0.59	7.08±0.45	38.98±4.99	m-sm
	L	5.79±0.97	7.32±0.29	37.51±3.27	
	C	5.23±0.75	7.27±0.31	39.24±3.19	
8	S	4.20±0.62	6.79±0.40	44.24±2.69	m
	L	5.66±1.05	7.13±0.34	44.21±4.34	
	C	5.14±0.79	7.13±0.32	43.05±3.30	
9	S	4.06±0.48	6.58±0.31	46.15+2.06	m
	L	5.51±1.16	6.92±0.35	42.62±3.69	
	C	4.96±0.69	6.89±0.21	44.73±3.32	
10	S	3.88±0.42	6.31±0.58	46.98±1.49	m
	L	4.98±0.96	6.28±0.52	42.28±4.50	
	C	4.69±0.65	6.53±0.25	43.60±2.47	
11	S	3.59±0.40	5.80±0.26	37.60±3.12	sm-m
	L	4.81±0.86	6.08±0.35	36.60±4.00	
	C	4.31±0.62	5.98±0.31	37.01±4.36	
12	S	3.04±0.48	4.91±0.28	32.19±3.50	sm
	L	3.89±0.71	4.91±0.34	28.78±3.03	
	C	3.30±0.49	4.60±0.45	28.80±2.34	
13	S	2.84±0.42	4.60±0.26	45.95±4.38	m
	L	3.07±0.42	3.91±0.26	42.78±2.47	
	C	2.81±0.40	3.92±0.37	42.77±3.08	
14	S	1.43±0.15	2.33±0.32	50.00±3.46	m
	L	1.81±0.18	2.35±0.44	42.91±4.55	
	C	1.16±0.10	1.62±0.24	43.07±1.63	

* m, metacentric; sm, submetacentric chromosome

**Fig 2:**
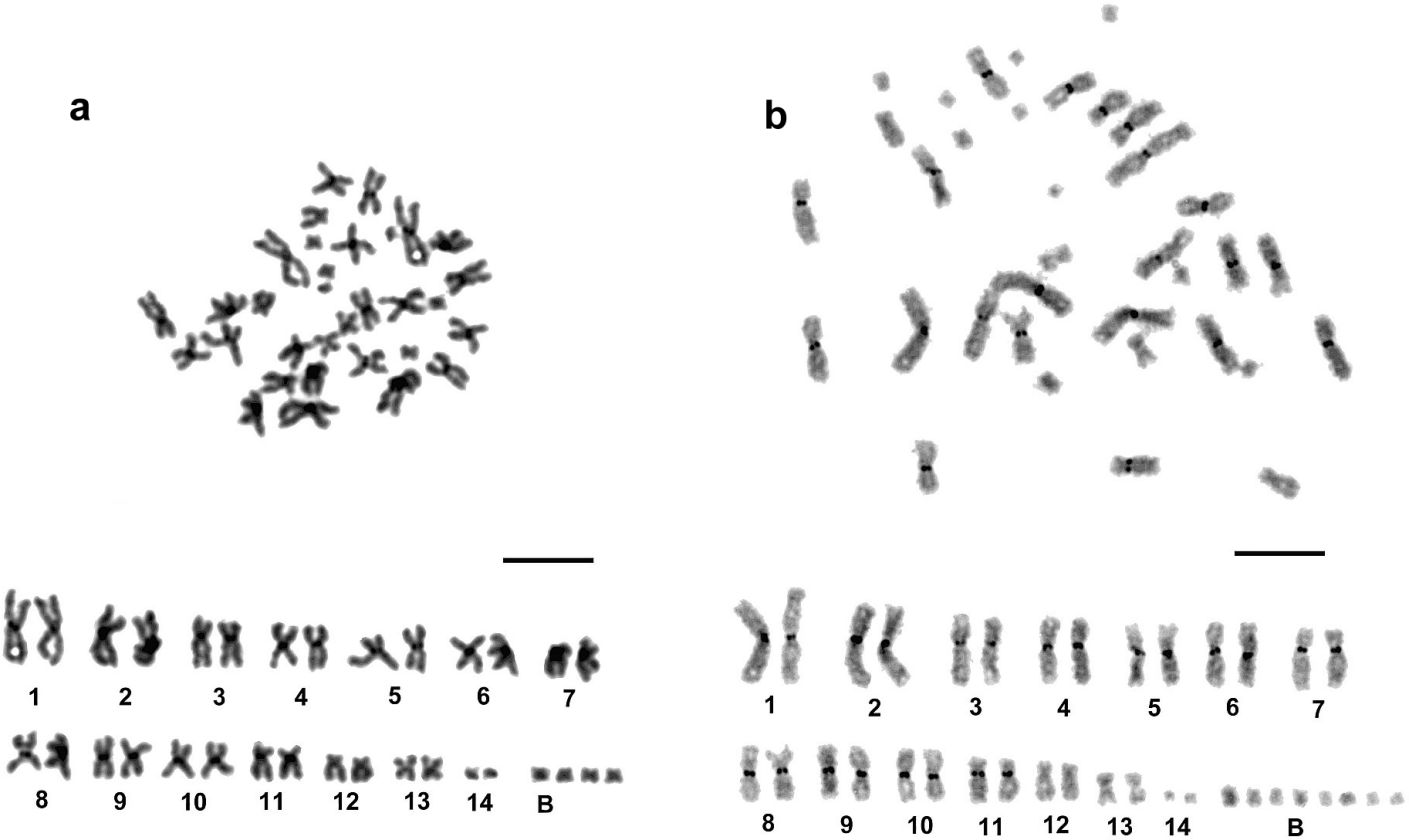
**Mitotic metaphases and respective karyotypes of *Sphaerium nucleus* with different numbers of B chromosomes: a, 2n = 28 + 4B, and b, C-banded chromosomes, 2n = 28 + 8B.** Scale bars = 10 μm.

Significant numbers of metaphase spreads with more than 28 chromosomes (from 14% to 35%) were observed in all three populations. The analysis of the corresponding karyotypes suggested the presence of a variable number of comparatively small (the mean length was 2.5 μm), biarmed, supernumerary (B) chromosomes, typically with 4 or 8 per cell (Fig 2A and 2B). In the Lithuanian population cells with 8 supernumerary chromosomes were found most often, but in the Slovakian population hyperdiploid cells contained either 4 or 8 B’s, and in the Czech population only cells with 4 supernumerary chromosomes were observed (see Table 1). Polyploid (4n) sets were rare; they were found in four cells (3 from Lithuanian and 1 from Czech population).

Results of the C-banding procedure were studied in five animals from different populations. Small but conspicuous pericentromeric constitutive heterochromatin blocks were always present on 11 chromosome pairs 1–11 (Fig 2B). Heterochromatin blocks were not observed (C-negative) on chromosome pairs 12, 13 and 14. B chromosomes also were C-negative.

### Karyotype of *Sphaerium corneum* var. *mamillanum*

**Fig 3:**
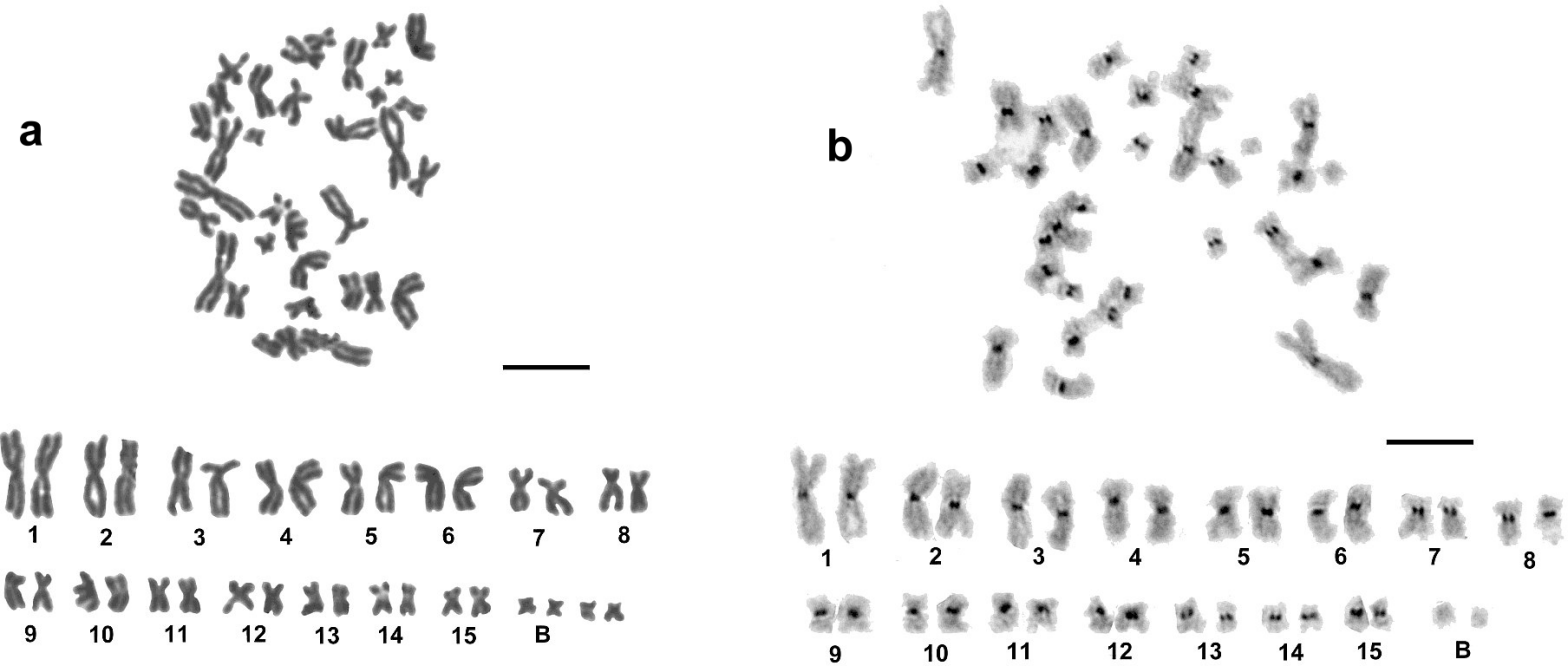
Chromosomes of *Sphaerium corneum* var. *mamillanum*: a, conventionally stained mitotic metaphase and karyotype, 2n = 30 + 4B, and b, C-banded mitotic metaphase and karyotype, 2n = 30 + 2B. Scale bars = 10 μm.

A total of 86 mitotic metaphase spreads from ten individuals were analysed. The modal diploid chromosome number was 2n = 30 (Table 1). A representative karyotype is shown in Fig 3A. The chromosomes ranged in size from 2.7 μm to 9 μm (Table 3). The TCL reached 75.56 μm. The karyotype consisted of all biarmed elements and, according to the centromere position, 13 chromosome pairs (pair number 1–3, 5, 6, 8–15) were classified as metacentric, and two pairs, 4 and 7, represented intermediates between the meta- and submetacentric structure. A comparative study of centromeric indexes and relative lengths revealed no significant (P<0.05) differences in the basic karyotype structure of this *S*. *corneum* var. *mamillanum* population from the population of *S*. *corneum* s. str., described in an earlier study [21].

**Table 3:** Measurements (mean±SD) and classification of modal diploid (A) chromosomes of *Sphaerium corneum* var. *mamillanum*.

Chromosome number	Absolute length (μm)	Relative length (%)	Centromeric index	Classification*
1	9.02±0.83	11.97±0.40	48.06±1.20	m
2	8.07±0.95	10.68±0.09	43.45±4.94	m
3	7.23±1.32	9.54±0.55	41.61±1.89	m
4	6.45±0.31	8.58±0.67	38.84±4.72	m-sm
5	6.25±1.67	8.19±1.18	44.06±2.76	m
6	5.47±0.56	7.25±0.17	43.12±3.52	m
7	4.56±0.55	6.04±0.03	40.25±5.26	m-sm
8	4.28±0.68	5.65±0.19	40.77±1.35	m
9	4.29±0.62	5.67±0.11	40.88±4.06	m
10	3.80±0.84	5.00±0.48	44.40±4.42	m
11	3.56±0.28	4.73±0.22	44.04±1.77	m
12	3.46±0.37	4.59±0.09	42.20±1.29	m
13	3.29±0.48	4.35±0.09	41.55±4.16	m
14	3.08±0.04	4.10±0.46	40.23±2.25	m
15	2.74±0.02	3.66±0.49	40.65±2.55	m

*m, metacentric; sm, submetacentric chromosomes.

Twenty-six of the 86 studied cells (30.23%) contained more than the modal number of chromosomes, with a maximum of 36. The supernumerary (B) chromosomes observed in these hyperdiploid cells were small biarmed elements and showed intra-individual variation from 0 to 6. The modal number of B’s, 4 per cell, was found in 18 (20.93%) of the studied metaphases (Fig 3A).

C-banding revealed that all of the chromosomes of the basic complement (A) showed a bright heterochromatic band in the centromeric region. No heterochromatin blocks were observed on any of the B chromosomes in the analysed metaphases (Fig 3B).

### Molecular and phylogenetic analyses

The 17 complete nucleotide sequences obtained during this study have been deposited in GenBank (Table 4, in bold). Partial sequences of mitochondrial 16S rDNA and nuclear ITS1 rDNA were determined for the specimens from Lithuania, Slovakia, Czech (S. Moravia), and Russia. Particular differences between sequences of related groups of *Sphaerium* spp. with pairwise deletion of gaps/missing data and inclusion of all substitutions (transitions and transversions) are indicated in the Tables 5 and 6. All individuals of *S*. *nucleus* from Lithuania, Slovakia, and Russia characterized for nuclear ITS1 rDNA sequences had identical genotypes. The individuals collected in Czech, however, were different from the *S*. *nucleus* collected in Lithuania, Slovakia, and Russia, as well as from the Ukrainian specimen sequenced by Lee & Ó Foighil [24]: the sequences differed by 4 bp and by 2 bp, respectively, in the ITS1 alignment of 556 sites (Table 5). Also, the ITS1 sequences of the *S*. *nucleus* with different genotypes have different lengths. The identical lengths of the ITS1 sequences of *S*. *corneum* and *S*. *solidum* were shorter than the ITS1 sequences of *S*. *nucleus*. All 16S sequences of *S*. *nucleus* from different populations have almost identical length but nucleotide differences varied from 6 bp to 13 bp (Table 6). In contrast, both of the sequences of *S*. *corneum* from Estonia were identical to *S*. *corneum* var. *mamillanum*.

**Table 4:** *Sphaerium* spp. subjected to molecular phylogenetic analysis with information of their host, locality and GenBank accession numbers.

Species	Locality	GenBank No and a source if it is not from this study*	
		16S	18S-ITS1-5.8S
*Sphaerium nucleus*	Ukraine	AY093573 [24]	AY093537 [24]
**Sphaerium nucleus**	Slovakia: a fen marsh near Vysna Pokoradz village	**HM208267, HM208268, HM208269**	**HM208261**
***Sphaerium nucleus***	Czechia: a marsh near Tvrdonice, South Moravia	**HM208271, HM208272, HM208273**	**HM208262**
***Sphaerium nucleus***	Russia: Moscow region	**HM208270**	**HM208263**
***Sphaerium nucleus***	Lithuania: Lake Terpežys	**HM208264, HM208265, HM208266**	**HM208260**
*Sphaerium corneum*	Germany	AF152037 [24]	AY093535 [24]
*Sphaerium corneum*	France: Rennes		AY093547 [24]
*Sphaerium corneum*	Lithuania: a pond in the North part of Vilnius	AY792316, AY792317 [21]	AY792319 [21]
*Sphaerium corneum*	Lithuania: River Vilnelė in Vilnius	AY792320 [21]	AY792321 [21]
***Sphaerium corneum***	Estonia: Lake Mustjarv	**GU128620, GU128621**	**KU863151**
***Sphaerium corneum***	Estonia: River Vaike-Emajogi	**GU128617**	**KU863152**
***Sphaerium corneum*, var. *mamillanum***	Estonia: stream between Lake Liinjarv and Lake Suurjarv	**GU128618, GU128619**	**KU863153**
*Sphaerium baicalense*	Russia: Lake Baykal		AY093534 [24]
*Sphaerium solidum*	Lithuania: Curonian Lagoon	FJ874903, FJ874904 [22]	GU123690 [22]
*Sphaerium solidum*	Hungary: Danube River	FJ874907, FJ874908, FJ874909 [22]	GU123689 [22]
*Sphaerium rhomboideum*	USA: Michigan	AF152038 [24]	AY093538 [24]
*Sphaerium occidentale*	USA: Michigan	AF152046 [24]	AY093542 [24]

*Sequences obtained in this study are marked in bold

**Table 5:** Average number of nucleotide differences between ITS1 dataset sequences of closest related groups of *Sphaerium* spp. with pairwise deletion of gaps/missing data and inclusion of all substitutions (transitions and transversions).

	Groups, sequence length	1.	2.	3.	4.
1.	*S*. *nucleus* (Ukraine), 550 bp				
2.	*S*. *nucleus* (Lithuania, Slovakia, Russia), 554 bp	4			
3.	*S*. *nucleus* (Czech), 556 bp	4	2		
4.	*S*. *corneum*, 542 bp	3	3	3	
5.	*S*. *solidum*, 542 bp	4	4	4	1

**Table 6:** Average number of nucleotide differences between 16S dataset sequences of closest related groups of *Sphaerium* spp. with pairwise deletion of gaps/missing data and inclusion of all substitutions (transitions and transversions).

	Groups,sequence length	1.	2.	3.	4.	5.	6.	Within groups
1.	*S*. *nucleus* (Ukraine), 475 bp							-
2.	*S*. *nucleus* (Lithuania, Russia), 475 bp	6						0
3.	*S*. *nucleus* (Slovakia), 475 bp	10	4					0
4.	*S*. *nucleus* (Czech), 474 bp	13	8	12				0
5.	*S*. *corneum* (2n = 36), 474 bp	9	9	13	14			0
6.	*S*. *corneum*, 474 bp	14.14	10.14	14.14	8.14	9.43		0.86
7.	*S*. *solidum*, 474 bp	13.29	9.29	13.29	7.29	12.29	5.14	1.43

**Fig 4:**
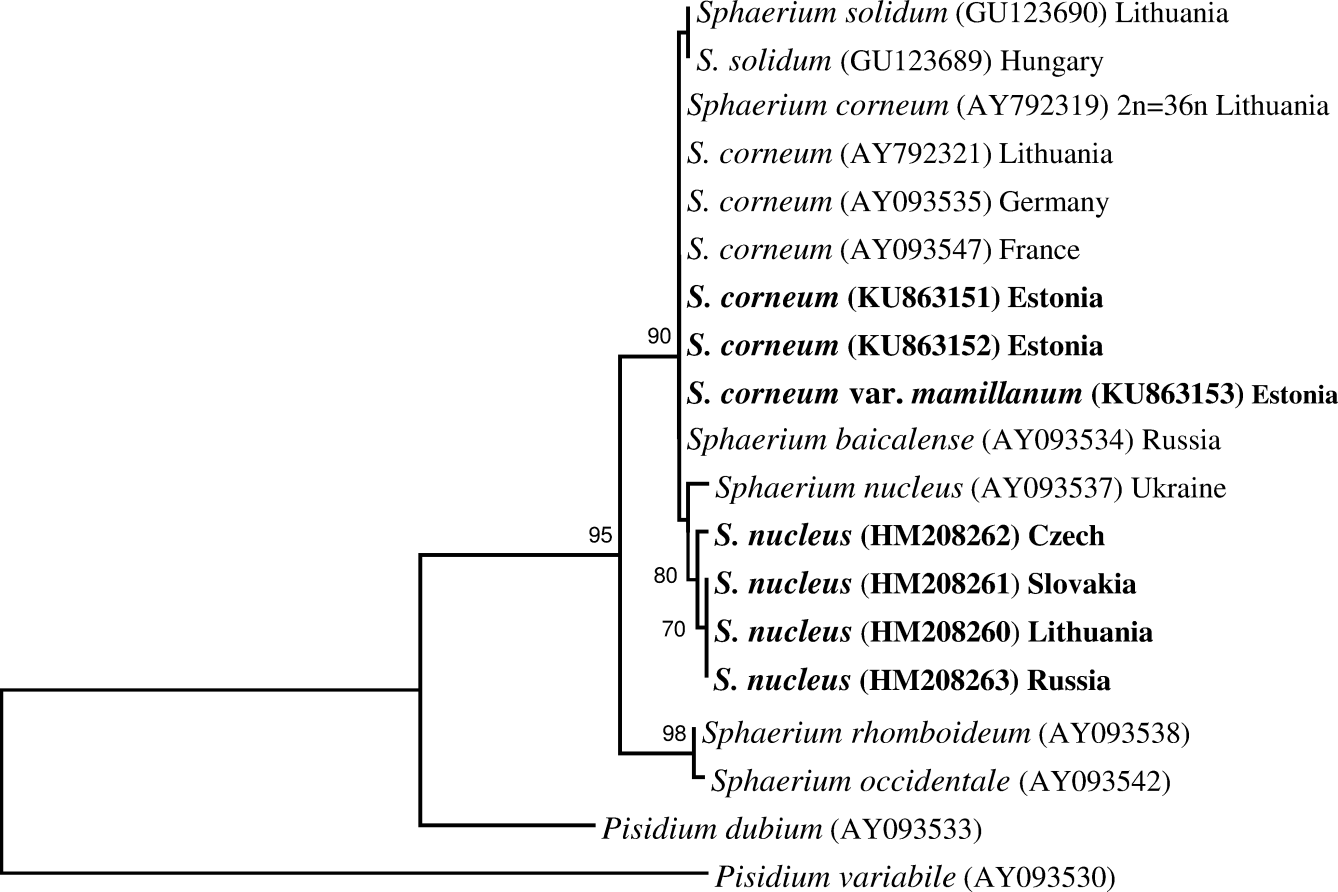
Phylogenetic tree obtained from ITS1 sequences of nuclear rDNA and based on the analysis of 520 sites. Bootstrap support given for maximum likelihood analysis (bootstrap replications = 1000, complete deletion of gaps/missing data). Bootstrap support values lower than 70% are not shown. Names of the target species are in bold. *Pisidium dubium* and *P*. *variable* were included as outgroups.

**Fig 5:**
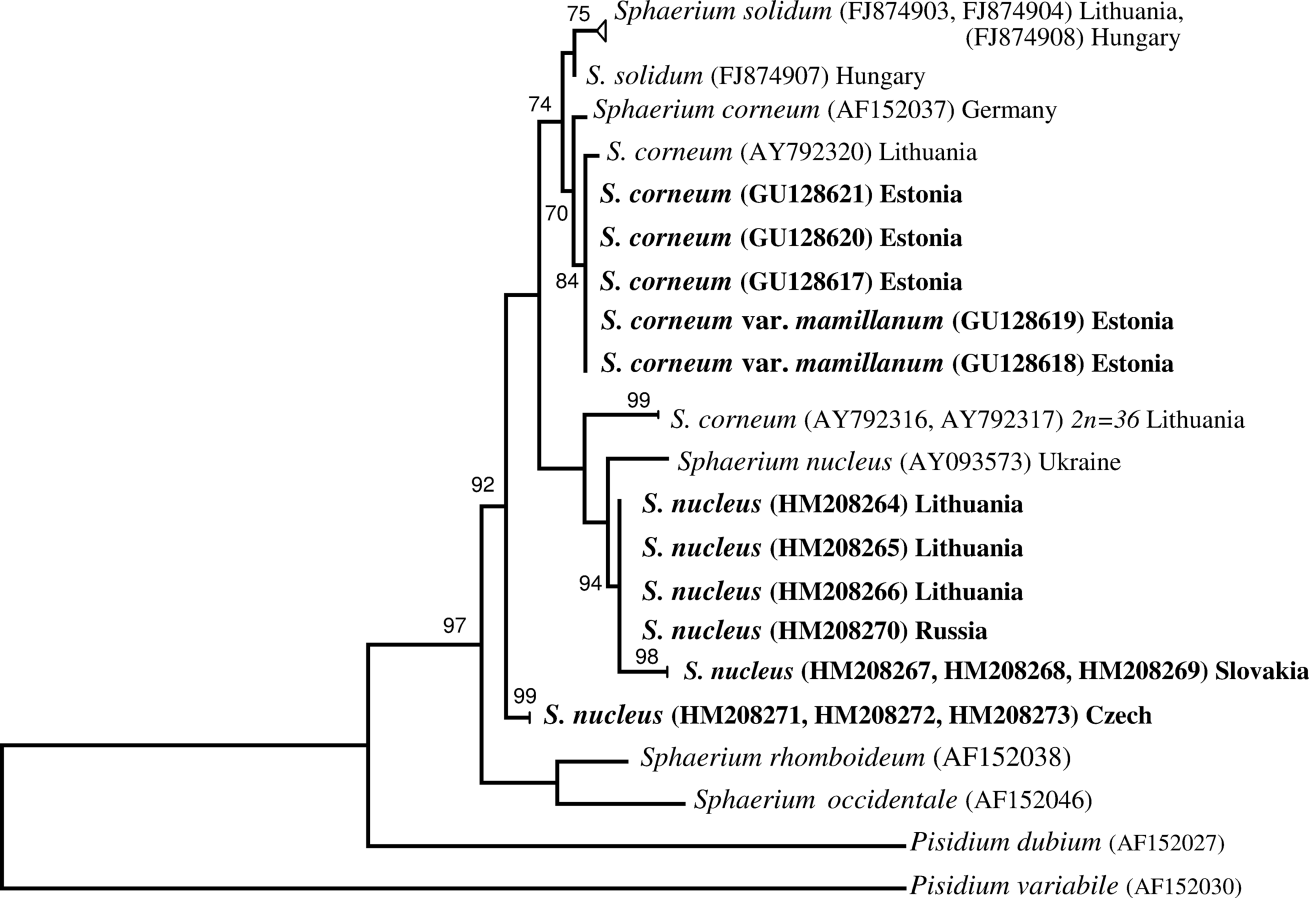
Phylogenetic tree for 16S haplotypes based on the analysis of 471 sites of mitochondrial 16S rDNA sequences. Bootstrap support given for maximum likelihood analysis (bootstrap replications = 1000, complete deletion of gaps/missing data). Bootstrap support values lower than 70% are not shown. Names of the target species are in bold. *Pisidium dubium* and *P*. *variable* were included as outgroups.

These two different sets of DNA sequences produced different tree topologies in the phylogenetic analyses (Figs 4 and 5). The genetically different groups of *S*. *nucleus* formed separated branches in both trees. In the ITS1 tree (Fig 4), all branches of *S*. *nucleus* clustered into one clade and the specimens from Czech, Lithuania, Slovakia, Russia, and Ukraine formed a subclade in a well­supported clade with *S*. *corneum*, *S*. *baicalensis*, and *S*. *solidum*. In the 16S tree (Fig 5), the Czech *S*. *nucleus* formed a distinct branch separated from all other *S*. *nucleus* and from a clade of *S*. *corneum* and *S*. *solidum* specimens.

## Discussion

While *S*. *nucleus* and *S*. *corneum* seem to possess only a few discriminative morphological characters, a comparative karyological analysis separates these species because they differ both in diploid numbers (2n = 28 and 2n = 30, respectively) and in the morphology and C-banding patterns of some chromosome pairs. On the other hand, although *S*. *corneum* var. *mamillanum* is a morphologically distinct form characterized by the presence of distinct embryonic shells on the umbones, our analysis revealed no significant differences (P<0.05) between the karyotype structure of this form and *S*. *corneum* s. str., previously studied by Petkevičiūtė et al. [21].

Conservatism in chromosome numbers is noticeable in the bivalve taxa listed by Nakamura [36] and Thiriot-Quievreux [37]. The evolution of unionid mussels has generally proceeded without change in chromosome number [38–39]. The first data on chromosome numbers for sphaeriid species were reported by Keyl [40], who found n = 18 and 2n = 36 in male meiosis of *S*. *corneum*. The subsequent rate of karyological descriptions in Sphaeriidae has been low. It is now known that the genus *Sphaerium* is characterized by an extreme karyotypic diversification, with mitotic chromosome numbers varying from 28 to 247 [19–22,41–43, this study]. *Sphaerium* could be considered a typical example of explosive speciation related to a high number of chromosomal reorganizations.

Most of the existing cytogenetic studies of sphaeriids have been focused on the number of chromosomes, and only 4 species have been studied for chromosome morphology [19,21–22]. The scarcity of comprehensive cytogenetic studies on sphaeriid species may be associated with the exceptionally high mitotic chromosome numbers found in most species analysed (see review in [19–20]). In addition, the presence of a variable number of supernumerary chromosomes was revealed in some species [21–22].

It is worth noting that all diploid sphaeriid species studied so far are representatives of the genus *Sphaerium*. Even in the diploid *Sphaerium* clams, karyotype composition varies from species to species, but different groups of species follow different patterns. Two species, *S*. *solidum* and *S*. *corneum*, have a stable karyotype morphology, with the same modal diploid chromosome number (2n = 30), a complement of biarmed metacentric and submeta-metacentric chromosomes of gradually decreasing size, and no significant interspecific karyotypic differences [22]. Nearctic species were regarded as highly polychromosomic, but Petkevičiūtė et al. [19] showed that *S*. *rhomboideum* is diploid, with 2n = 44.

As described in this study, *S*. *nucleus* has the lowest chromosome number (2n = 28) of the sphaeriids studied to date. Karyotypes with low chromosome numbers, 2n = 28 and 2n = 30, are exclusively composed of biarmed meta- and submetacentric chromosomes, while uni-armed telo- and subtelocentric chromosomes are present in the karyotype of *S*. *rhomboideum* (2n = 44) and in the karyotypic form (2n = 36) from species group of *S*. *corneum* [19,21]. Differences in the number and morphology of chromosomes lend support to the assumption that Robertsonian translocations are involved in the cytogenetic divergence of species. Reduction in chromosome numbers by Robertsonian rearrangements was previously suggested in the marine bivalve families Mytilidae and Pectinidae [44–45].

The second karyological peculiarity of all studied European species of *Sphaerium* s. str. is the presence of mitotically unstable B chromosomes. *Sphaerium rhomboideum* differs in this regard because no B chromosomes have been found in its cells [19]. B chromosomes of *Sphaerium* spp. are small metacentric elements; in the cells of *S*. *corneum* and *S*. *solidum* they are significantly smaller than any of the basic (A) chromosomes, while in *S*. *nucleus* Bs are larger than the smallest chromosomes of the basic set. Furthermore, different degrees of numerical stabilization and interpopulation differences in frequency of B chromosomes were revealed. B chromosomes of *S*. *corneum* and *S*. *solidum* showed a more dispersed distribution, varying from 0 to 10, and from 0 to 6, respectively, but the even number of Bs (mostly four or eight) was more often observed than any odd number [21–22]. During this study, eight B chromosomes were commonly observed in the Lithuanian population of *S*. *nucleus*, while four or eight Bs were present with approximate frequencies in cells of clams from Slovakia, and four Bs were recorded in hyperdiploid cells in the Moravian population.

The occurrence and persistence of B chromosomes in a lineage probably has a genomic explanation and is thus of evolutionary significance [46]. B chromosomes are an intriguing class of chromosomes. They are additions to the standard (A) chromosome complement and follow their own evolutionary pathway. The term B chromosomes include very heterogeneous types of chromosomes; their only consistent feature is that they are not essential for survival of an individual and are present in some individuals from some populations in some species [46–48]. Data for B chromosomes of bivalve mollusc species are very scarce. Variable numbers of B chromosomes were recorded in clonal lineages of marine clams of the genus *Lasea* (Veneroida) [49–50]. Presence of 1–3 small supernumerary chromosomes was observed in *Cerastoderma edule* (Veneroida) and they were presumed to be B chromosomes [51]. Later analysis using restriction enzyme banding demonstrated that those B chromosomes were, in fact, the result of chromosomal fission involving the largest submetacentric chromosome pair [52].

For *S*. *nucleus*, the C-banding technique showed heterochromatic (C-positive) regions near the centromeres of chromosomes pairs 1–11, but no C-blocks were revealed in chromosomes pairs 12–14 of the main complement. All B chromosomes were C-negative in the *S*. *nucleus* samples. In the karyotype of *S*. *corneum* var. *mamillanum*, conspicuous C-positive regions were revealed in all chromosomes of the main complement and, as with *S*. *nucleus*, the B chromosomes were all C-negative. B chromosomes are heterochromatic in many organisms, but they can be C-negative as well [48,53]. In most animals heterochromatin is detected at the pericentromeric region [54]. C-banding analyses in marine bivalves *Ostrea denselamellosa*, *O*. *angasi*, *O*. *conchaphila*, *Mytilus edulis*, *M*. *galloprovincialis*, *M*. *trossulus* and *Crassostrea angulata* indicated that pericentromeric heterochromatin is not common in these species, although telomeric and interstitial heterochromatin is [55–60]. Supposedly, the karyotypes with higher telomeric heterochromatin must have an older phylogenetic status [57].

The results of our study show that *S*. *corneum* s. str. and form *mamillanum* share identical ITS1 and 16S sequences. Both the karyological and the molecular evidence fail to support the independent taxonomic status of *S*. *corneum* var. *mamillanum*. While the karyological analysis of *S*. *nucleus* in this study revealed the same basic karyotype structure for representatives of three populations, the comparisons of the ITS1 and 16S sequences indicated the different lineages within this species. The phylogenetic analyses and differences in the 16S sequences clearly separated Czech *S*. *nucleus* from the other populations studied. Also, there was a clear divergence in 16S between samples from the Lithuanian, Russian populations and Slovakian population, and *S*. *nucleus* from Ukraine. DNA sequence analyses of *S*. *solidum* showed that only one site was different from ITS1 of *S*. *corneum* [22]. Moreover, ITS1 was found to be identical in *S*. *corneum* and endemic of Lake Baikal *S*. *baicalense* [24]. The ITS1 differences among *S*. *nucleus* samples are more significant: 2–4 bp and the different lengths of ITS1 in all three lineages of *S*. *nucleus*. So, in the context of the Evolutionary Species Concept, one could treat the three lineages of *S*. *nucleus* as three good species.

Morphological and molecular studies of sphaeriid phylogeny are incongruent (see [61]). At the species level, however, *S*. *corneum* and *S*. *nucleus* represent closely related sister taxa, both morphologically and in molecularly based studies (see [8,24]). In the morphologically based analysis of Korniushin & Glaubrecht [8], five *Sphaerium* species (*S*. *corneum*, *S*. *solidum*, *S*. *nitidum*, *S*. *nucleus*, and *S*. *rhomboideum*) form a monophyletic group recognized as *Sphaerium* s. str. Recent karyological and molecular studies [22] confirmed the close relationships of *S*. *corneum* and *S*. *solidum*. Although the molecular data did not support the placement of North American *S*. *rhomboideum* as sister to European *S*. *nucleus* and strongly suggested that *S*. *rhomboideum* be reassigned to the subgenus *Herringtonium* [24], recent karyological analysis [19] gave an unexpected result–the chromosome set of *S*. *rhomboideum* is diploid. So, the morphologically based intergeneric division of *Sphaerium* species is correlated with karyotypic patterns; in the *Sphaerium* s. str. group, all karyologically studied species have diploid chromosome sets, including Palaearctic *S*. *corneum*, *S*. *solidum*, *S*. *nucleus*, and Nearctic *S*. *rhomboideum*.

Freshwater habitats have relatively discrete boundaries, suggesting that populations of freshwater invertebrates should also be discrete [62]. Furthermore, ecological peculiarities of sphaeriid clams, together with their odd system of reproduction, could lead to a low rate of genetic exchange and to manifestation of founder effect followed by formation of highly isolated populations. Regarding their reproduction, sphaeriids appear as specialized freshwater molluscs, being simultaneous hermaphrodites and viviparous–they broods embryos up through the juvenile stage in the suprabranchial chamber [6,25,63–65]. Even a single individual can give origin to a distinct and often isolated population. *Sphaerium nucleus* lives in small, often temporal water bodies, so, considerable changes in population size and rapid differentiation of populations under dissimilar selective regimes is predictable. Ecological heterogeneity may have been a key-factor responsible for genetic divergence [66], but the genetic identity of the *S*. *nucleus* specimens from the Slovakian, Lithuanian, and Russian populations do not correlate with ecological or ecotypic similarity. The genetic divergence in *S*. *nucleus* is unlinked to any apparent pattern of karyological and morphological variation or ecological preference. This highlights a disconnection between molecular, karyological and morphological evolution. Our findings demonstrate that reliance on the current morphological taxonomy underestimates the underlying genetic diversity. The increasing availability of DNA sequences and utilization of molecular markers in taxonomic and phylogenetic studies reveal that a broad spectrum of taxa contains sets of morphologically similar, but genetically distinct, lineages [67]. The use of a genetic yardstick, however, might be problematic. The number of methods available for delimiting species markedly increases in recent years and different approaches to species delimitation exist [68–71] however each have a unique set of challenges [70], so they must be used with caution. It is difficult to calibrate the minimum threshold of divergence to establish interspecific separations between organisms with inadequate taxonomies, such as sphaeriid bivalves, which are also characterised by odd systems for reproduction, extraordinary dispersal abilities and populations commonly found in isolated unstable environments. According to the estimation of experts, the inferences drawn from species delimitation studies should be conservative [25,70–71] and it is better refer to monophyletic groups as lineages than falsely delimit ‘species’. A plausible and acceptable statistical method to recognise cryptic species in sphaeriid bivalves has not been applied and still doesn’t exist. Moreover, all haplotypes of *S*. *nucleus* share the same basic karyotype structure and there are no karyotypic barriers (meiotic constrains) for interbreeding of individuals with distant haplotypes, or as indicated Rannala [71], genetic isolation alone does not prove that the lineages are incapable of interbreeding, and referring on the Biological Species Concept (the requirement of reproductive incompatibility between species) such lineages do not represent actual species. We hope that data on *Sphaerium* species diversity could be useful in creating a statistical method able to recognise cryptic species and, herewith, do not fail to separate genetically closely related species, such as *S*. *corneum* and *S*. *solidum*.

In general, studies that incorporate molecular, morphological and/or karyological data will provide much better descriptions and interpretations of biological diversity than those that focus on just one approach. Considering the genetic diversity uncovered in the *S*. *nucleus* complex within the limited range studied here, it is likely that more cryptic diversity is present. Our data show that many questions about this complex of species remain to be answered.
